# Variants Affecting the C-Terminal of CSF1R Cause Congenital Vertebral Malformation Through a Gain-of-Function Mechanism

**DOI:** 10.3389/fcell.2021.641133

**Published:** 2021-03-19

**Authors:** Bowen Liu, Sen Zhao, Zihui Yan, Lina Zhao, Jiachen Lin, Shengru Wang, Yuchen Niu, Xiaoxin Li, Guixing Qiu, Deciphering Disorders Involving Scoliosis and COmorbidities (DISCO) study, Terry Jianguo Zhang, Zhihong Wu, Nan Wu

**Affiliations:** ^1^Department of Orthopedic Surgery, State Key Laboratory of Complex Severe and Rare Diseases, Peking Union Medical College Hospital, Peking Union Medical College and Chinese Academy of Medical Sciences, Beijing, China; ^2^Beijing Key Laboratory for Genetic Research of Skeletal Deformity, Beijing, China; ^3^Graduate School of Peking Union Medical College, Beijing, China; ^4^Medical Research Center, Peking Union Medical College Hospital, Peking Union Medical College and Chinese Academy of Medical Sciences, Beijing, China

**Keywords:** colony stimulating factor 1 receptor, congenital vertebra malformation, C-terminal variant, gain-of-function mechanism, zebrafish model

## Abstract

*CSF1R* encodes the colony-stimulating factor 1 receptor which regulates the proliferation, differentiation, and biological activity of monocyte/macrophage lineages. Pathogenic variants in *CSF1R* could lead to autosomal dominant adult-onset leukoencephalopathy with axonal spheroids and pigmented glia or autosomal recessive skeletal dysplasia. In this study, we identified three heterozygous deleterious rare variants in *CSF1R* from a congenital vertebral malformation (CVM) cohort. All of the three variants are located within the carboxy-terminal region of CSF1R protein and could lead to an increased stability of the protein. Therefore, we established a zebrafish model overexpressing *CSF1R*. The zebrafish model exhibits CVM phenotypes such as hemivertebral and vertebral fusion. Furthermore, overexpression of the mutated *CSF1R* mRNA depleted of the carboxy-terminus led to a higher proportion of zebrafish with vertebral malformations than wild-type CSF1R mRNA did (*p* = 0.03452), implicating a gain-of-function effect of the C-terminal variant. In conclusion, variants affecting the C-terminal of CSF1R could cause CVM though a potential gain-of-function mechanism.

## Introduction

Colony-stimulating factor 1 receptor (CSF1R) regulates the proliferation, differentiation and biological activity of monocyte/macrophage (Mϕ) lineages (Stanley et al., 1997). *CSF1R* is also expressed in Paneth cells, renal proximal tubule epithelial cells, and placental trophoblasts, indicating a pleiotropic role of CSF1R protein in embryonic development (Arceci et al., 1989, 1992; Pixley and Stanley, 2004; Huynh et al., 2009; Menke et al., 2009). The CSF1R protein includes an extracellular ligand-binding domain and an intracellular tyrosine kinase domain (PTK domain) (Pixley and Stanley, 2004). The extracellular domain of CSF1R binds to ligands such as CSF1 and IL34, which further induces autophosphorylation and protein dimerization (Wang et al., 2012; Stanley and Chitu, 2014). Downstream molecules interact with the intracellular part of CSF1R and are phosphorylated by the PTK domain. The phosphorylated signal molecules could activate the downstream signaling pathway, promoting the proliferation and differentiation of hematopoietic precursor cells, especially mononuclear phagocytes. Heterozygous deleterious variants in *CSF1R* have been reported to cause adult-onset leukoencephalopathy with axonal spheroids and pigmented glia (ALSP, MIM: 221820) (Oosterhof et al., 2019), a neurological disease characterized by executive dysfunction, memory decline, personality changes, motor impairments, and seizures (Svensson et al., 2011; Rodríguez-Tornos et al., 2016; Makrythanasis et al., 2018). Notably, most of the reported ALSP-related heterozygous pathogenic variants were located at the intracellular PTK domain (Oosterhof et al., 2019).

More recently, homozygous variants in *CSF1R* have been linked to brain abnormalities, neurodegeneration, and dysosteosclerosis (MIM: 618476), which is characterized by progressive neurologic deterioration and sclerotic bone dysplasia (Guo et al., 2019; Oosterhof et al., 2019). *Csf1r*^−*/*−^ mouse model resembled the skeletal phenotypes of the patients carrying bi-allelic *CSF1R* variants, implicating the important role of *CSF1R* in bone development (Erblich et al., 2011). Furthermore, an abnormal vertebral arch compared to normal individuals was observed in the zebrafish model with biallelic loss-of-function mutations in *csf1ra* and *csf1rb*, indicating the potential effect of *CSF1R* variants on vertebral morphology (Oosterhof et al., 2019).

In this study, we analyzed variants in *CSF1R* in a cohort of congenital vertebral malformation (CVM), which has been partially attributed to genetic defects previously (Wu et al., 2015, 2019; Chen et al., 2016, 2020; Liu et al., 2019; Yang et al., 2019, 2020; Lin et al., 2020; Ren et al., 2020). *In vitro* and *in vivo* functional experiments were then performed to explore the effect of these variants on protein expression and vertebral morphology.

## Methods

### Human Subjects

Five hundred and eighty-three patients diagnosed with CVM were consecutively enrolled and collected in the cohort between 2009 and 2018 at Peking Union Medical College Hospital, as a part of the Deciphering Disorders Involving Scoliosis and COmorbidities (DISCO) study (http://www.discostudy.org/). Detailed phenotypic data was recorded. X-ray, computed tomography (CT), and magnetic resonance imaging (MRI) were also taken. Exome sequencing and bioinformatic analysis were conducted as described previously (Zhao et al., 2021). Variant interpretation was then performed based on the genome Aggregation Database (gnomAD, http://gnomad.broadinstitute.org/). Rare variants in *CSF1R* were extracted and filtered with the following criteria: (1) truncating (non-sense, frameshift, splice acceptor/donor) and minor allele frequency (MAF) ≤ 0.001 or (2) missense variants absent from public databases in the general population.

### Sanger Sequencing

Candidate variants of *CSF1R* identified in our cohort were validated by Sanger sequencing. The variant-encoding amplicon was amplified by PCR from genomic DNA obtained from patients and purified using an Axygen AP-GX-50 kit (lot no. 05915KE1). Sanger sequencing was then performed on an ABI3730XL instrument.

### Plasmid Construction

The pEGFP-C1-based vector was used for the construction of wild-type *CSF1R* and mutated *CSF1R* (NM_005211.3: c.2906_2909dupATCA, c.2797G>T, c.2749_2758delGACAGGAGAG) plasmids. All plasmids were verified by DNA sequencing.

### Cell Culture, Transfection, and Western Blotting Analysis

Cos-7 cells were maintained in Dulbecco's Modified Eagle's medium (DMEM, Invitrogen) supplemented with fetal bovine serum (Gibco), penicillin (50 U/ml), and streptomycin (50 μg/ml) in six-well plates. The full-length wild-type or mutant CSF1R constructs (plasmid pEGFP-C1 1 μg) was transfected into the cells, respectively. After 48 h, western blotting was used to assess the protein expression level in the cell lysates with the following antibodies: rabbit anti-human CSF1R monoclonal antibody (1:1,000, Abcam, ab229188) and mouse anti-GAPDH monoclonal antibody (1:1,000, ZSJQB Co., Ltd.).

### Zebrafish Husbandry and Fertilization

Tg (Ola.Sp7:nlsGFP) transgenic zebrafish, where GFP expression was driven by an Sp7 promoter in osteoblasts, was utilized for animal model establishment. The zebrafish were kept at 28°C and fed with brine shrimp twice a day. From the 2–4-cell stage to 3 days post fertilization (dpf), zebrafish were exposed to methylene blue which inhibited fungal contamination.

### *In vitro* mRNA Transcription

A human wild-type *CSF1R* DNA sequence was cloned into the PCS2+ plasmid, constructing a human wild-type *CSF1R* plasmid. Then, an indel variant (c.2749_2758delGACAGGAGAG) was constructed to generate a human-mutated *CSF1R* plasmid containing a mutated gene sequence. After the linearization of plasmids, transcription was performed with mMESSAGE mMACHINE™ SP6 Ultra Transcription Kit (Ambion) to obtain corresponding mRNA.

### *In vivo* mRNA Overexpression Experiment

Human *CSF1R* mRNA (50 pg for each), i.e., the wild-type mRNA or the mutated mRNA, was dissolved into distilled water (2 nl for each) and injected into embryos at the 1–2-cell stage, respectively. An equal amount of water was injected into the embryos, which were fertilized as the control group. All the individuals in the experimental groups and control group were maintained for phenotype evaluation.

### Fluorescence Imaging and Phenotype Evaluation

Fluorescence images were collected at 14 dpf by fluorescent microscopy. The vertebral morphology was observed and recorded for phenotypic evaluation and statistical analysis.

### Genetic Analysis of CSF1R-Associated Genes

Twenty genes which are biologically linked to CSF1R (*IL34, CSF1, SOS, GRB2, INPP5D, INPPL1, PIK3R1, PIK3R2, SOCS1, SOCS3, CBL, FYN, GRAP2, LYN, RASA1, SHC11, THOC5, TES1, CBL, PLCG2, SLA2*) were selected for analysis according to public databases including Pubmed (https://pubmed.ncbi.nlm.nih.gov/) and Online Mendelian inheritance in Man (OMIM, https://www.omim.org/). Further, variant filtration of those genes was performed according to the same interpretation method as we did for *CSF1R*.

### Statistics

Statistical differences between different experiment groups and the control group were evaluated with the Chi-square test. All statistical procedures were carried out using GraphPad Prism 8. ^*^, ^**^, ^***^, and ^****^ denote *p* < 0.05, < 0.01, < 0.001, and < 0.0001, respectively.

## Results

### Pathogenic Variants Identified in CSF1R

**Figure 1:**
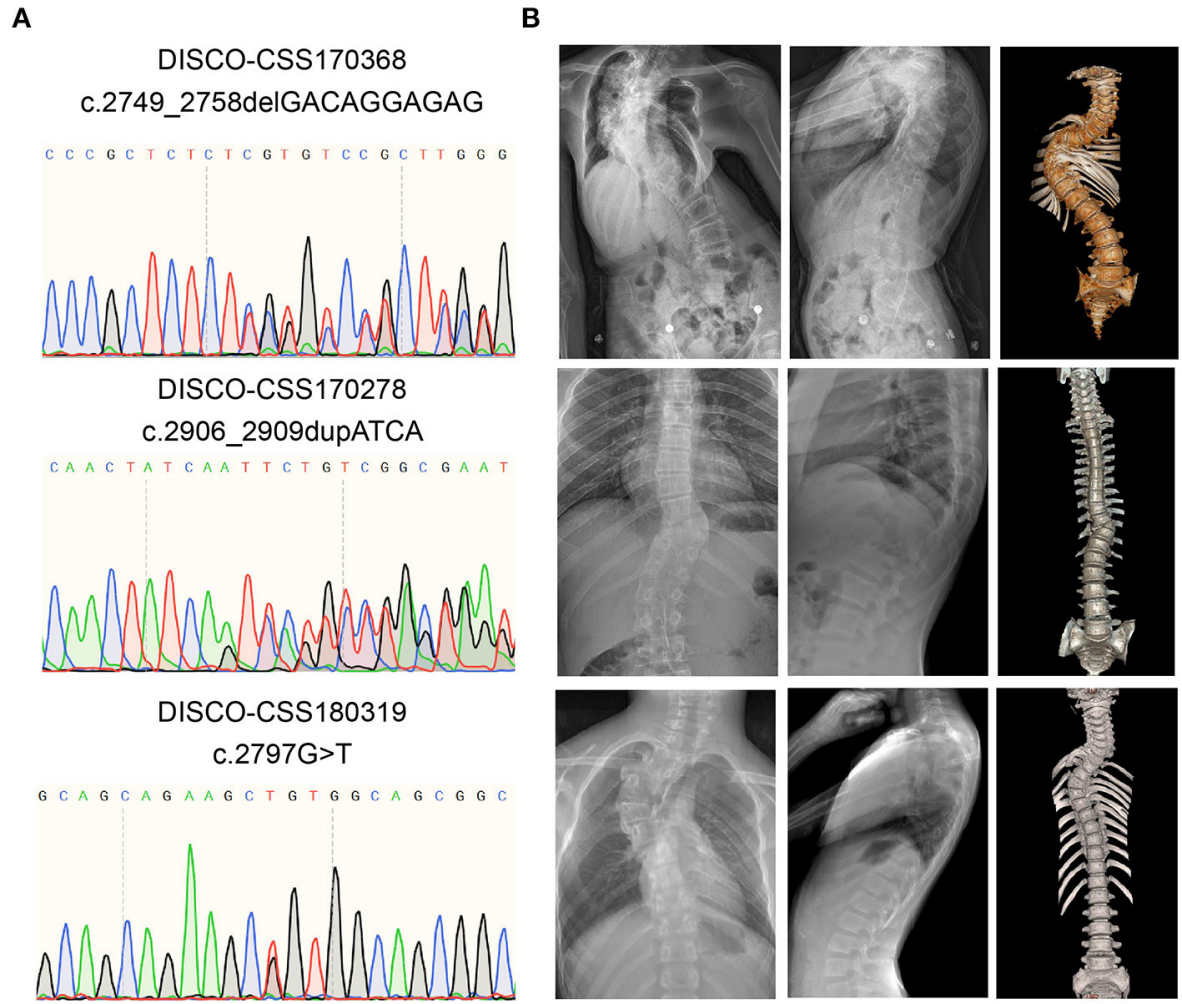
Variant information and clinical phenotype of *CSF1R* carboxy-terminal variants in three patients with CVM. **(A)** Results of Sanger sequencing of the rare *CSF1R* carboxy-terminal variants. **(B)** Spinal plain radiograph and computed tomography (CT) scan three-dimensional reconstruction of those CVM patients. CVM, congenital vertebral malformation.

**Figure 2:**
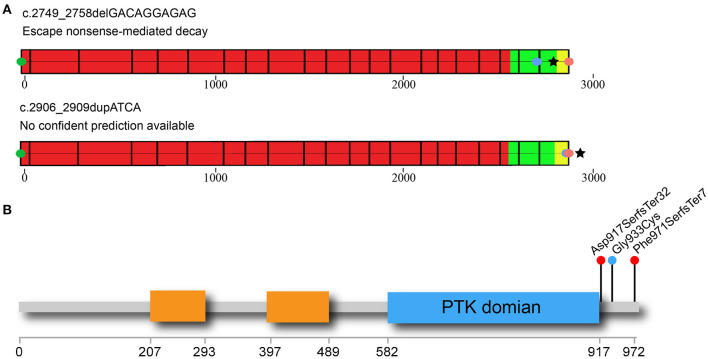
Non-sense-mediated decay (NMD) prediction and distribution of rare *CSF1R* C-terminal variants in CVM patients. **(A)** NMD prediction of two *CSF1R*-truncating variants identified in the CVM cohort. Green circle indicates the CDS start site; red circle indicates the canonical site of CSF1R protein; blue circle indicates the location of the truncating variant. Black star symbol indicates the predicted termination site of the mutated protein. The red part of protein-coding exons indicates the region in which truncating variants lead to NMD. On the contrary, the alleles with truncating variants located in the green part of protein could escape from NMD. The variants located in the yellow region of the protein may lead to non-stop RNA decay. One of the C-terminal *CSF1R* variants in the CVM cohort (c.2749_2758delGACAGGAGAG) is predicted to escape NMD. The other variant (c.2906_2909dupATCA) located within the yellow region could generate a new stop codon in the 3′ UTR region of mRNA, adding several amino acids on the C-terminus of the CSF1R protein (p.Phe971SerfsTer7). Hence, this variant allele is also predicted to escape from NMD. **(B)** Simplified diagram of the CSF1R protein is shown in the figure, with important domains and deleterious variants indicated. The immunoglobin domains are indicated by orange rectangles. The intracellular protein tyrosine kinase (PTK) domain is indicated by the blue rectangle. The truncating variants identified in DISCO-CSS170368 and DISCO-CSS170278 are indicated by the red circles. The missense variant identified in DISCO-CSS180319 is indicated by the blue circle. CVM, congenital vertebral malformation.

From three patients with CVM, three deleterious heterozygous variants in *CSF1R* were identified, including two predicted truncating variants and one novel missense variant (Table 1). DISCO-CSS170368 was a 14-year-old girl admitted for spinal surgery (Figure 1A). The spinal plain radiograph showed that the coronal Cobb angle of the main curve (T2–L1) was 114°. The CT scan and three-dimensional reconstruction showed segmentation defects of T5–T9, dysplasia of T3–9, L4 hemivertebra, and left 6th and 7th rib abnormality (Figure 1B). MRI indicated mild diastematomyelia and syringomyelia. The girl showed no dyspnea, numbness or weakness of the limbs, backache or extremity pain, or marfanoid symptoms during the course of the disease. The clinical diagnoses of this patient were severe congenital scoliosis, pulmonary dysfunction, and diastematomyelia along with syringomyelia. WES identified a heterozygous truncating *CSF1R* variant (NM_005211.3: c.2749_2758delGACAGGAGAG), which was then confirmed by Sanger sequencing (Figure 1A). This variant is absent from public databases and our in-housed database encompassing 849 exome data (Table 1). Ten nucleotides were deleted between positions 2749 and 2758 in exon 21, resulting in a frameshift mutation in *CSF1R*. The mRNA harboring this mutation is predicted to escape from non-sense-mediated mRNA decay (NMD) by the NMD Esc Predictor (Figure 2A) (Coban-Akdemir et al., 2018). Hence, this frameshift mutation could consequently generate a truncated protein product (p.Asp917SerfsTer32).

**Table 1:** Deleterious and rare *CSF1R* variants identified in patients with congenital vertebral malformation.

**Patient**	**Ref transcript**	**Variant nomenclature**	**Mutation type**	**gnomAD allele** ** frequency**	**gnomAD allele** ** count**	**In-house** ** frequency**	**In-house count**	**CADD**	**Polyphen-2** ** HDIV score**
DISCO-CSS170368	NM_005211.3	c.2749_2758delGACAGGAGAG	Frameshift variant	0	0	0	0	NA	NA
		(p.Asp917SerfsTer32)							
DISCO-CSS180319	NM_005211.3	c.2797G>T	Missense variant	0	0	0	0	11.22	0.981
		(p.Gly933Cys)							
DISCO-CSS170278	NM_005211.3	c.2906_2909dupATCA	Frameshift variant	0.00003254	10	0	0	NA	NA
		(p.Phe971SerfsTer7)							

DISCO-CSS180319 was a 10-year-old female patient with CVM. Imaging examination revealed a 56° Cobb angle of the main curve (T3–T7). Besides, a structure disorder of T3–T7 was also observed in CT scan and three-dimensional reconstruction (Figure 1B). A missense variant (NM_005211.3: c.2797G>T) was identified in this girl (Figure 1A) and was also absent from the public and in-house databases. This variant is predicted to be deleterious by Polyphen-2 (Polyphen2 HDIV score: 0.981).

DISCO-CSS170278 was a 16-year-old boy with thoracic and lumbar scoliosis. The spinal plain radiograph revealed the presence of T10 hemivertebra (Figure 1B). WES analysis revealed a deleterious variant (NM_005211.3: c.2906_2909dupATCA) in *CSF1R*, which has been reported in the East Asian population with low frequency (0.0005) (Figure 1A). This deleterious variant could lead to a reading-frame shift. As a result, the last two amino acids of CSF1R were deleted and six extra amino acids were added to the C-terminus of the protein.

Interestingly, all of the three rare *CSF1R* variants identified in our cohort were located within or near the carboxy-terminal region downstream of the PTK domain (Figure 2B), which has been reported to be responsible for protein degradation mediated through the ubiquitination–proteasome pathway (Pawson, 1995; Schlessinger, 2000). No pathogenic variant in this region has ever been reported to be related to human diseases. Hence, those newly identified deleterious *CSF1R* variants might contribute to CVM through a distinct mechanism, which remains to be further investigated.

### Variants Affecting the C-Terminal of CSF1R Increased the Stability of Protein and Could Induce Vertebral Malformation in Zebrafish

**Figure 3:**
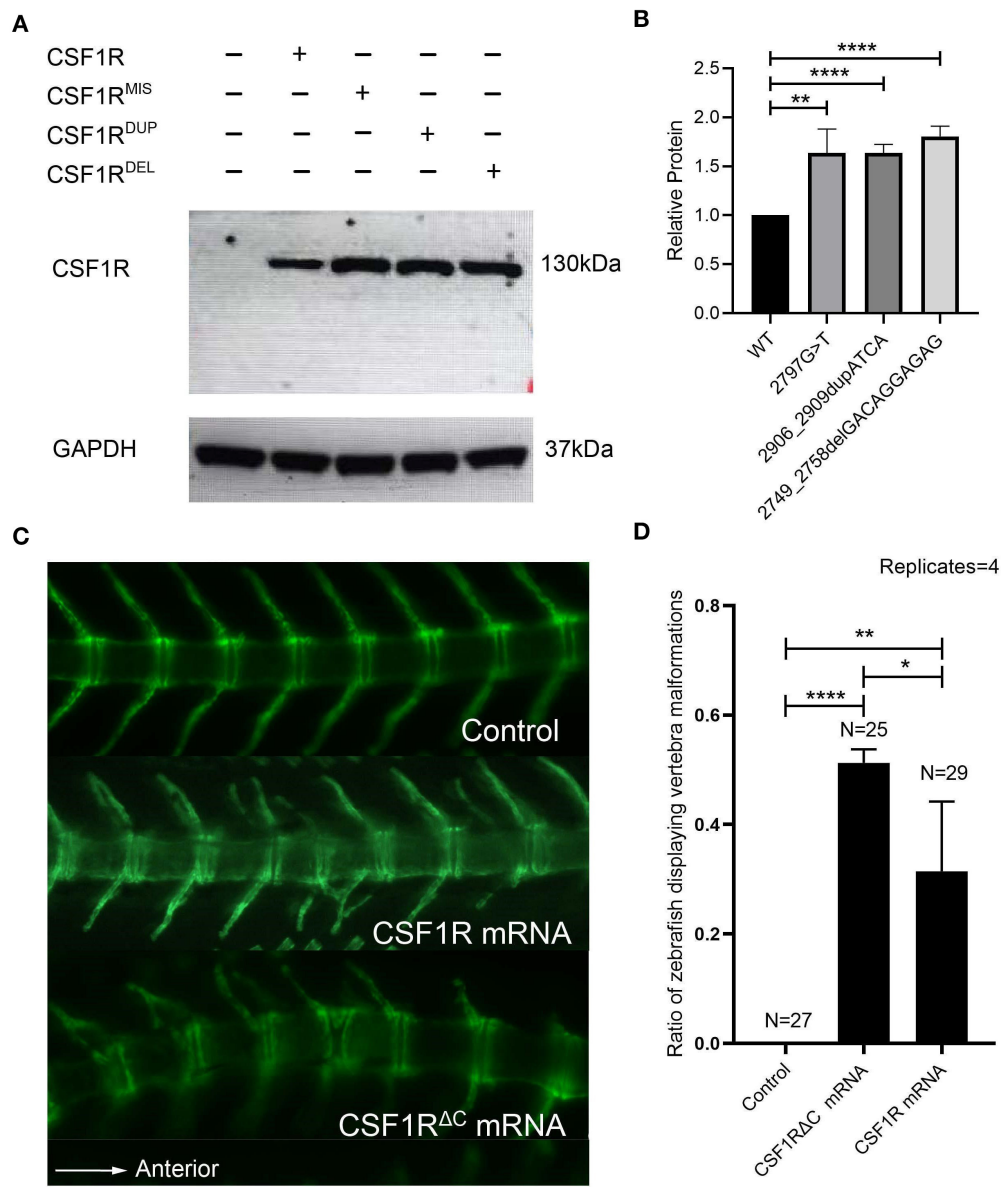
*In vitro* and *in vivo* functional study of *CSF1R* C-terminal variants. **(A)** Human CSF1R protein in total cell lysates made from cos-7 cells transfected with wild-type or mutant CSF1R constructs. GAPDH is used as a loading control. CSF1R^MIS^: c.2797G>T; CSF1R^DUP^: c.2906_2909dupATCA; and CSF1R^DEL^: c.2749_2758delGACAGGAGAG. **(B)** Quantification of relative wild-type and mutant CSF1R protein levels (*n* = 3). Protein levels of mutants were significantly increased compared to wild type (c.2797G>T, *p* = 0.00259; c.2906_2909dupATCA, *p* = 0.00005; c.2749_2758delGACAGGAGAG, *p* = 0.00004). Protein levels were normalized to GAPDH, and CSF1R was set to 1. **(C)** Fluorescence imaging (side view, anterior to the right) of Tg(Ola.Sp7:nlsGFP) transgenic zebrafish injected with 50 pg of wild-type *CSF1R* or *CSF1R*^Δ*C*^ human mRNA at 21 days post fertilization (dpf). The transgenic zebrafish injected with distilled water are fertilized and imagined as the control group. Note the vertebra labeled with green fluorescence. **(D)** A significant difference of the percentage of zebrafish displaying CVM phenotypes exists between experimental groups (*CSF1R* mRNA: 24%, 7/29; *CSF1R*^Δ*C*^ mRNA: 52%, 13/25) and control group (0%) (Chi-square test, *CSF1R* mRNA: *p* = 0.00635; *CSF1R*^Δ*C*^ mRNA: *p* = 0.00002). Compared to the zebrafish injected with *CSF1R* mRNA in the period of embryo, the zebrafish injected with *CSF1R*^Δ*C*^ mRNA have a significantly higher percentage of vertebral malformation phenotypes (Chi-square test *p* = 0.03452). Error bars represent one SD, *n* = 4 replicates. A different clutch of zebrafish was observed for each replicate. *p < 0.05; **p < 0.01; ***p < 0.001; ****p < 0.0001; CVM, congenital vertebral malformation.

To determine the functional consequence of the C-terminal *CSF1R* variants *in vitro*, we transfected wild-type and mutant CSF1R constructs into cos-7 cells and examined the cellular levels of protein products with Western blotting. As a result, all of the three mutants accumulated at greater levels than the wild type, suggesting increased stability of mutant CSF1R proteins (Figures 3A,B).

Therefore, we proposed that the heterozygous deleterious *CSF1R* variants might exert a gain-of-function effect on CSF1R biological function through disturbing the carboxy-terminal region of the protein. We then overexpressed the mutated *CSF1R* alleles (NM_005211.3: c.2749_2758delGACAGGAGAG) which is depleted of all the carboxy-terminal regions downstream of the PTK domain in the zebrafish model. This mutated mRNA-deleted carboxy-terminal region is referred to as *CSF1R*^Δ*C*^ mRNA in the following text.

The human *CSF1R* and *CSF1R*^Δ*C*^ mRNAs were injected into Tg (Ola.Sp7:nlsGFP) zebrafish embryos, respectively. Fluorescence images of the spine were collected at 21 dpf by fluorescent microscopy. The zebrafish of the *CSF1R* mRNA overexpression group exhibited vertebral malformations including vertebral fusion, hemivertebra, and fused vertebral arches (Figure 3C), which recapitulated the human CVM phenotype. These phenotypes indicate that the dose effect of *CSF1R* expression might be related to vertebral malformation. Intriguingly, injection of human *CSF1R*^Δ*C*^ mRNA led to a higher proportion of zebrafish with vertebral malformations than the wild-type mRNA did (*p* = 0.03452) (Figure 3D). These results revealed that the carboxy-terminus-mutated *CSF1R* alleles might exert gain-of-function effects and thereby cause vertebral malformations.

### Mutational Spectrum in Genes Interacting With CSF1R

As an essential signaling transductor, CSF1R participates in several biological processes in individual development and interact with many other genes. To explore whether the variants in CSF1R-related genes may also be related with clinical phenotypes like vertebral abnormalities, we identified 21 genes of which the protein products interacted with CSF1R (*IL34, CSF1, SOS, GRB2, INPP5D, INPPL1, PIK3R1, PIK3R2, SOCS1, SOCS3, CBL, FYN, GRAP2, LYN, RASA1, SHC11, THOC5, TES1, CBL, PLCG2, SLA2*) and performed a genetic spectrum analysis. Twelve potential deleterious rare variants were identified in different patients, including four truncating variants and eight missense variants (Table 2). Among those CSF1R-related genes, *CBL* encodes an adaptor protein c-Cbl for CSF1R. It positively regulates CSF1R ubiquitination in a manner dependent on its variant SH2 and RING finger domains, then marking active CSF1R for degradation (Pawson, 1995; Schlessinger, 2000). One of the binding sites of c-Cbl on CSF1R is 969aa, which lies in the carboxy-terminal region of CSF1R where all three pathogenic variants identified in *CSF1R* were located (Wilhelmsen et al., 2002). We prioritized two heterozygous *CBL* variants, including a rare truncating variant and a novel missense variant, from two patients in our CVM cohort. Those deleterious variants of CBL may affect the interaction between CSF1R and c-Cbl and lead to CVM by a similar mechanism as carboxy-terminal region variants of CSF1R do.

**Table 2:** Deleterious and rare variants in genes interacting with *CSF1R*.

**Gene symbol**	**Ref transcript**	**Variant nomenclature**	**Mutation type**	**gnomAD allele frequency**	**gnomAD allele count**	**In-house frequency**	**In-house count**	**CADD**
*CBL*	NM_005188.3	c.2435-2A>G	Splice acceptor variant	0.0000122	3	0	0	NA
	NM_005188.3	c.640C>T(p.Pro214Ser)	Missense variant	0	0	0	0	17
*SLA2*	NM_032214.3	c.-43-1G>T	Splice acceptor variant	0.000004103	1	0	0	NA
*GRB2*	NM_002086.4	c.652dupT(p.Ter218LeufsTer32)	Frameshift variant	0	0	0	0	NA
*IL34*	NM_001172772.1	c.247G>T(p.Ala83Ser)	Missense variant	0	0	0	0	24.3
	NM_001172772.1	c.352T>A(p.Tyr118Asn)	Missense variant	0	0	0	0	16.25
*FYN*	NM_153047.3	c.779C>T(p.Ala260Val)	Missense variant	0	0	0	0	19.28
*INPPL1*	NM_001567.3	c.1294A>C(p.Asn432His)	Missense variant	0	0	0	0	21.9
*THOC5*	NM_001002877.1	c.43C>T(p.Arg15Ter)	Stop gain variant	0	0	0	0	NA
	NM_001002877.1	c.655A>G(p.Ile219Val)	Missense variant	0	0	0	0	29.9
	NM_001002877.1	c.235G>A(p.Asp79Asn)	Missense variant	0	0	0	0	26.3
*SOCS1*	NM_003745.1	c.77C>T(p.Ser26Phe)	Missense variant	0	0	0	0	17.6

## Discussion

In this study, we used an exome-level genomic approach to identify potentially pathogenic variants of *CSF1R* genes in a CVM cohort. We identified three rare deleterious heterozygous C-terminal *CSF1R* variants, which were proved to increase the stability of protein in *vitro*. The CVM patients carrying those *CSF1R* variants exhibited vertebral malformations and early onset of scoliosis clinically. The zebrafish overexpression model resembled those vertebral phenotypes of CVM patients, indicating a gain-of-function mechanism.

In previous studies, *CSF1R* has been related to ALSP, which was inherited in an autosomal dominant pattern (Rademakers et al., 2011). ALSP is a fatal neurological disease characterized by progressive cognitive and motor impairment and seizures. The majority of the reported variants related with ALSP are missense or in-frame indels within the PTK domain (Guo et al., 2019). Recent studies indicated that bi-allelic *CSF1R* variants contribute to skeletal abnormalities in an autosomal recessive pattern (Guo et al., 2019; Oosterhof et al., 2019). The skeletal phenotypes of affected patients were referred to as the DOS-Pyle disease spectrum, which is characterized by sclerotic skull and flat and diffusely dense vertebral bodies (Guo et al., 2019). The pleiotropic effects of *CSF1R* could be attributed to the different inheritance modes and the variety of variants. Unlike the reported pathogenic *CSF1R* mutations, the variants identified in our cohort were located within the carboxy-terminal region of protein. This terminal region contains two autophosphorylation sites (923aa and 969aa), which offer binding sites for c-Cbl and participate in the degradation of CSF1R (Wilhelmsen et al., 2002; Ho et al., 2020). We proposed that the biological function of the protein could be affected when the carboxy-terminal region is mutated, which contributes to different clinical phenotypes like vertebral malformation.

Apart from the variety of variants and different pathogenic mechanisms, the age of onset is another factor which potentially affects the clinical symptoms of *CSF1R* variant carriers. The average age of symptom onset for ALSP is about 40 years (Sundal and Wszolek, 1993; Adams et al., 2018), while the average age of the patients with *CSF1R* deleterious variants identified in our cohort is around 10 years. The CVM patients may be too young to exhibit neurological phenotypes, which makes vertebral malformation-related scoliosis the only clinical symptom of those patients.

As an indispensable regulator of the monocyte/macrophage lineage, *CSF1R* has already been studied in variable animal models with the assistance of the transgenic technique. For instance, bi-allelic *Csf1r* deficiency in mouse was reported to cause brain development abnormalities and sclerosing skeletal dysplasia which leads to death within 6 weeks (Dai et al., 2004). Besides, the zebrafish model with biallelic loss-of-function mutations in *csf1ra* and *csf1rb* was also established in recent studies. This *csf1r*^*DM*^ *(csf1ra*^−*/*−^*, csf1rb*^−*/*−^*)* zebrafish also exhibited neurological abnormalities primarily, along with skeletal phenotypes like osteopetrosis (Oosterhof et al., 2019). Considering the gain-of -function effect of the newly identified heterozygous *CSF1R* variants, the overexpression experiment could be used to prove the pathogenicity of the *CSF1R* mono-allelic mutations identified in patients with spine abnormalities like CVM.

Furthermore, we focused on the detailed structural and biological effects of the variants and hypothesized how these variants affected the biological function of CSF1R. One of the *CSF1R* variants (NM_005211.3: c.2749_2758delGACAGGAGAG) identified in our cohort was related to higher protein expression level and led to more severe clinical phenotypes as compared with that of the other two deleterious variants. This variant (NM_005211.3: c.2749_2758delGACAGGAGAG) deletes the entire carboxy-terminus region sequence (p.Asp917SerfsTer32), which is important for the binding of c-Cbl and thus the ubiquitination-mediated protein degradation. However, the other two *CSF1R* variants only lead to minor structural disturbance of the protein carboxy terminus and consequently confer milder effects on the protein expression and human orthopedic manifestations. This phenomenon indicates a genotype–phenotype correlation in *CSF1R*-related CVM.

Correspondingly, considering the potential role c-Cbl plays in the etiology of *CSF1R*-related CVM, we also focused on this protein and identified two deleterious rare *CBL* variants in our cohort. It is demonstrated that c-Cbl binds to activated CSF1R and mediates its degradation through the tyrosine-kinase binding (TKB) domain (Wilhelmsen et al., 2002). Interestingly, the novel deleterious CBL missense variant (NM_005188.3: c.640C>T) identified in our CVM patient is located within the TKB domain exactly, which potentially interrupts the interaction between c-Cbl and CSF1R, further affecting CSF1R degradation. Besides, another variant (NM_005188.3: c.2435-2A>G) of CBL is predicted to affect the splicing process of c-Cbl and create a critical structure disorder, which could also inhibit c-Cbl biological function. All those CBL deleterious variants were identified in CVM patients, who exhibited similar clinical phenotypes as CSF1R carboxy-terminus variant carriers did. This phenomenon indicates that the CSF1R ubiquitination and degradation might be one of the important biological processes related to CVM. The inhibition of CSF1R degradation may contribute to the enhancement of CSF1R biological function, further affecting bone metabolism and vertebral development, leading to vertebral malformation.

Besides, the variants in our cohort may also affect the biological function of CSF1R through generating structural disorders potentially. For instance, the missense variant (NM_005211.3: c.2797G>T) leads to amino acid change (p.Gly933Cys), for which glycine was replaced by cystine. Glycine is a relatively stable amino acid with low molecule weight. The amino acid is replaced by cystine, which is much more active and easily oxidizable. The oxidized cystine tends to form a disulfide bond with each other, potentially promoting the formation and stabilization of the CSF1R homodimer. The biological activity of protein could be enhanced by such structure disorder, which contributes to vertebral malformation. All the hypothesized mechanisms mentioned before remains to be explored further.

In conclusion, our study reveals the pathogenicity of heterozygous carboxy-terminal variants of *CSF1R* in CVM. The variants affect the biological function of the *CSF1R* product through potential gain-of-function effects.

## Data Availability Statement

The datasets generated during the current study are available in the Mendeley repository, https://data.mendeley.com/datasets/wzjxyz99st/1.

## Ethics Statement

The studies involving human participants were reviewed and approved by ethics committee at Peking Union Medical College Hospital (JS-098). Written informed consent to participate in this study was provided by the participants' legal guardian/next of kin. The animal study was reviewed and approved by ethics committee at Peking Union Medical College Hospital (JS-098). Written informed consent was obtained from the individual(s), and minor(s)' legal guardian/next of kin, for the publication of any potentially identifiable images or data included in this article.

## Author Contributions

NW, ZW, TZ, BL, SZ, and GQ conceived of the project and designed the study. NW, BL, SZ, ZY, SW, and YN recruited the patients and collected and interpreted the data. BL and SZ conducted the statistical analysis and bioinformatic analyses. BL and JL performed the *in vivo* experiments. BL, LZ, and XL performed the *in vitro* experiments. BL, SZ, and NW wrote the first draft of the manuscript and critically revised the work for important intellectual content. All authors contributed to the article and approved the submitted version.

## Conflict of Interest

The authors declare that the research was conducted in the absence of any commercial or financial relationships that could be construed as a potential conflict of interest.
